# The Role of Short-Chain Fatty Acids and Altered Microbiota Composition in Autism Spectrum Disorder: A Comprehensive Literature Review

**DOI:** 10.3390/ijms242417432

**Published:** 2023-12-13

**Authors:** Piotr P. Lagod, Saleh A. Naser

**Affiliations:** Division of Molecular Microbiology, Burnett School of Biomedical Sciences, College of Medicine, University of Central Florida, 4110 Libra Drive, Orlando, FL 32816, USA; piotr.lagod@ucf.edu

**Keywords:** autism spectrum disorder, short-chain fatty acids, propionic acid, gut microbiota, gut-brain axis, microbiome shift

## Abstract

Autism spectrum disorder (ASD) is a complex neurodevelopmental condition characterized by deficits in communication and social interactions, restrictive and repetitive behavior, and a wide range of cognitive impediments. The prevalence of ASD tripled in the last 20 years and now affects 1 in 44 children. Although ASD’s etiology is not yet elucidated, a growing body of evidence shows that it stems from a complex interplay of genetic and environmental factors. In recent years, there has been increased focus on the role of gut microbiota and their metabolites, as studies show that ASD patients show a significant shift in their gut composition, characterized by an increase in specific bacteria and elevated levels of short-chain fatty acids (SCFAs), especially propionic acid (PPA). This review aims to provide an overview of the role of microbiota and SCFAs in the human body, as well as possible implications of microbiota shift. Also, it highlights current studies aiming to compare the composition of the gut microbiome of ASD-afflicted patients with neurotypical control. Finally, it highlights studies with rodents where ASD-like symptoms or molecular hallmarks of ASD are evoked, via the grafting of microbes obtained from ASD subjects or direct exposure to PPA.

## 1. Introduction

Autism spectrum disorder (ASD) is a complex neuro-developmental condition characterized by deficits in communication and social interactions, repetitive and restrictive behavior, and a wide range of comorbidities, especially those involving the gastrointestinal (GI) tract [1,2,3]. ASD is often diagnosed in early childhood (as early as 2 years of age); however, milder cases of autism may be diagnosed later in life [4,5]. In the Diagnostic and Statistical Manual of Mental Disorders, the following are classified under ASD: autistic disorder, pervasive developmental disorder not otherwise specified (PDD-NOS), and Asperger syndrome [6]. Reports published by the Centers for Disease Control and Prevention (CDC) from the Autism and Developmental Disabilities Monitoring Network, which surveys the prevalence of ASD among eight-year-old children, show that the prevalence of ASD tripled in the last 20 years. In the year 2000, the prevalence was 1 in 150 children, whereas in the report for the surveillance year of 2018 (published in 2021), the prevalence of ASD was 1 in 44 children [7]. This could also be considered a significant increase from the previous surveillance year (2016), in which the prevalence was 1 in 54 children. The prevalence of ASD is four times higher in males than in females [7]. The striking rise in prevalence, alongside the paucity of available treatments, urges an intensification of research into ASD’s etiology, treatment approaches, and possible means of prevention [3]. Additionally, ASD brings significant economic and social burden on society and families with children afflicted with ASD and significantly lowers their quality of life [8]. 

Although some of the increase in prevalence can be attributed to changes in diagnostic criteria and increased awareness of both parents and health, evidence also shows that environmental factors also contribute to the increase [9,10]. For instance, the rise in prevalence is significantly outpacing the changes in population genetics [11], alongside evidence from monozygotic twins where the concordance is not 100% [12], suggesting that ASD arises from an interplay of complex genetic and environmental factors. A significant amount of evidence points to factors such as toxins, metabolic abnormality, oxidative stress, and immune dysregulation during the gestational period [13,14]. There is increasing evidence demonstrating a link between ASD and the GI tract, in which as much as 70% of ASD patients exhibit GI symptoms including diarrhea, gastroesophageal reflux, abnormal epithelial barrier permeability, immune dysregulation, and significant GI inflammation [13,15,16,17]. In addition to GI comorbidities, several studies show significant alteration of the microbiota composition versus age-matched control patients not exhibiting ASD symptoms, in which the former are characterized by an increased abundance of short-chain fatty acid (SCFA) producers [18,19,20]. Amid SCFAs, propionic acid (PPA) is of highest interest, as there has been an increase in recent evidence showing its link to ASD development and symptom severity. 

As suggested by recent findings, a shift in microbiota and elevated levels of SCFAs, in particular PPA, might be among the environmental factors contributing to the development of ASD. This comprehensive review aims to provide a reader with a background on the role of SCFAs in the human body, alongside the role of gut microbiota. It also provides a summary of the current studies aiming to establish the difference in composition of microbiota in ASD-afflicted patients versus age-matched neurotypical controls. Finally, it provides an overview of recent rodent studies where PPA or direct microbiota transfer from ASD patients cause ASD-like symptoms in rodents.

Publications relevant to this study were identified with the aid of PubMed using key words that include autism (or ASD) + microbiota, dysbiosis, bacteria, short-chain fatty acid, and propionic acid. Only studies within the last 20 years were included. Moreover, only studies involving proper age-matched controls were considered. The ages of participants in the review studies were 1 to 18 years old.

## 2. Short-Chain Fatty Acids

### 2.1. Classification and Sources

Short-chain fatty acids (SCFAs) are monocarboxylic acids with fewer than six carbon atoms [21,22]. Amongst the SCFAs, the most prominent, constituting 95% of all SCFAs present in the human intestine, are acetic acid (AA), propionic acid (PPA), and butyric acid (BA) [23]. Their structures are comprised of a carboxylic acid moiety and a short unbranched hydrocarbon chain containing two, three, or four carbons, respectively [24]. The total concentration of SCFAs in the human large intestine reaches 50–200 mM [25], with a molar ratio of 60:20:20 for acetic, propionic, and butyric acids, respectively. 

Most of the SCFAs present in the large intestine result from the fermentation of carbohydrates by various species of bacteria (which will be discussed in subsequent chapters), along with a smaller contribution from undigested peptides in the small intestine [26]. Starches are among the most prominent substrates for SCFA production. Although they are usually digested in the small intestine by pancreatic amylase, some starches can remain undigested or partially digested (collectively called resistant starch (RS)) and move into the large intestine to be a substrate for bacterial fermentation [27]. Their resistivity to digestion can be attributed to physical inaccessibility (as in partially milled grain and legumin), being trapped in granules with high amylose content (amylose is much harder to break down enzymatically than amylopectin), chemical modification (processed food), and retrogradation (temperature fluctuation causing changes in starch properties) [28,29]. Other fermentable carbohydrates include dietary fibers, which are part of the plant cell wall, and can be classified as either water soluble or water insoluble. The former are highly fermentable and significantly contribute to the production of SCFAs; the latter, although fermentable to a much lower extent, increase the volume of fecal matter and lower the time of colonic transit [30]. Examples of fermentable fibers include cellulose, hemicellulose, and pectin [27]. Amino acids such as valine, leucine, and isoleucine could serve as a less prominent substrate for SCFA production [31]. 

Although the majority of SCFAs present in the intestine are produced by resident bacteria, SCFAs are present in food, either naturally occurring or added during food processing, as PPA has antifungal properties [3]. During the production process, food with naturally occurring SCFAs usually undergo fermentation by various types of bacteria; food especially rich in PPA and/or BA include cheese and butter [32]. Baked goods (especially those with a long shelf life), dried fruits, and other processed foods contain PPA and its salts, which include potassium propionate, sodium propionate, and calcium propionate, as food preservatives [33]. Although the microbiota are the main contributor to high levels of SCFAs, diet can also play a role.

### 2.2. Metabolism and Distribution

The most prominent SCFAs in the human intestine (i.e., acetic acid, propionic acid, and butyric acid) are found at around 50–200 mM concentration in the intestinal lumen. However, based on a seminal study involving sudden-death victims, the concentration of SCFAs is 1000-fold lower in the portal vein with values reaching ~375 μM, further falling to ~140 μM in hepatic blood, and finally reaching ~79 μM in peripheral blood [26]. However, the evaluation of the concentration and utilization of SCFAs in the human body is challenging, as only a few studies involving sudden-death victims were conducted [34]. Additionally, the concentration of SCFAs both in the intestine and circulation is highly dependent on the individual’s diet (the uptake of SCFAs naturally present in food or added as a preservative), intestinal microbiota (specific microorganism species and producers of a high amount of SCFAs and their subtypes), and the individual’s metabolism [23,32,33]. 

Each of the major SCFAs have distinct distribution and are differentially processed in the body. Butyric acid is mainly absorbed through monocarboxylate transporters utilized by colonocytes as an energy source [35,36], while acetic and propionic acids are transported to the liver via the portal vein. In the liver, a large portion of propionic acid is metabolized, while the remaining portion travels through various tissues via blood circulation. SCFAs can readily cross the blood–brain barrier (BBB) and exert an effect on the central nervous system (CNS) [35,37]. Although the exact mechanism of SCFAs’ influence on the CNS remains largely unknown, the growing number of animal studies demonstrate that SCFAs can affect subject behavior and influence important neurological processes at the molecular level [38]. 

In a seminal in vitro study, it was also shown that treatment of neural stem cells with PPA significantly shifts their differentiation faith. While in the control, the neural stem cells differentiated to an equal ratio of neurons and glia cells, in the PPA-treated cells, the shift in differentiation resulted in 80% of cells being positive for glial markers and only 20% for neuronal markers [3]. Similarly, an increase in glial cells has been observed in the post mortem brain of ASD patients [39]. This finding suggests that, during early fetal development, PPA may perturb neural patterning and brain development. Additionally, PPA-treated cells exhibit signs of neuroinflammation, as evidenced by an increase in pro-inflammatory cytokine levels [3].

### 2.3. SCFA Levels in ASD

Several recent studies reported an increase in SCFAs in the stool of ASD subjects versus age-matched neurotypical controls. For instance, He et al. reported this in a study involving 40 ASD children with constipation (matched to 40 neurotypical controls). In addition to the change in the microbiome, as summarized in table in the subsequent chapter, they reported a significant increase in the concentration of PPA. Other SCFAs (AA and BA) were elevated; however, they were not statistically significant [40]. Corretti et al. reported a significant increase in PPA and BA, while De Angelis et al. reported a significant increase in PPA and AA [41,42]. Wang et al. also reported that AA, PPA, BA, and isobutyric acid were elevated in ASD patients. Additionally, patients with propionic acidemia are often diagnosed with ASD. In this condition, high levels of propionic acid are present in the circulation, due to an inefficiency in propionyl-CoA carboxylase activity [43]. More research is needed on the levels of PPA and other SCFAs in the ASD population, as most of the studies mainly focused on the microbiome shift without consideration of SCFAs. However, across microbiota studies, species that are consistently elevated are SCFA producers [42,44].

## 3. Microbiota

### 3.1. Overview

The human body is colonized by a myriad of microorganisms (with estimates ranging from 3.8 × 10^16^ to 1 × 10^14^) [45,46], which inhabit the skin as well as the mucosal cavities. The number of genes encompassed in the microbial community vastly outnumbers the human genome. Although throughout the years, the role of the microbiome in human health and disease was not sufficiently applicated, in the past few decades, with the advent of modern gene sequencing and advanced bio information tools alongside growing evidence of an altered microbiome in a multitude of diseases, the human microbiome became a center of intensive investigation [35]. The GI tract is the most densely populated, with trillions of bacteria, fungi, and viruses collectively called microbiota and accounting for approximately 1 kg of human gut weight [38,47]. Bacteria phyla that are mostly found in human feces include Firmicutes, Bacteroidetes, Actinobacteria, and Proteobacteria (collectively comprising up to 90% of the total bacteria found), with the addition of Verrucomicrobia and Fusobacteria, which are found at a lower abundance. Based on the presence of some microbes in the placenta, amniotic fluid, and meconium, it is believed that microbial colonization of the GI tract begins in the prenatal period [48] and continues during birth, breastfeeding, skin contact, and the introduction of various foods. Additionally, the mode of child delivery influences the population of bacteria that initially colonizes a newborn’s GI tract. Children born vaginally are initially colonized by their mother’s fecal and vaginal bacteria, while those born via cesarean delivery are initially colonized by bacteria found on the skin and in the hospital environment [49]. For instance, 75% of the fecal microbiota of vaginally born children is related to the fecal microbiota of their mothers; however, children born via c-section only exhibit 41% similarity [50]. Another factor contributing to microbial composition is gestational age, where premature infants lack specific bacteria genera typically found in full-term infants [51]. The human microbiome and its host usually interact in a mutually beneficial manner; the host provides a stable environment and food source for the microorganisms, while they help process indigestible food, synthesize important nutrients and vitamins, influence the immune system, and minimize the growth of harmful organisms though niche competition [35,52,53]. In addition to providing processed nutrients, microorganisms produce a variety of compounds that can influence the host organism in the GI tract and beyond. 

### 3.2. Microbiota Shift in Children with ASD

Numerous recent studies have shown alteration in the microbiota of children diagnosed with ASD versus age-matched neurotypical controls [54,55]. The table below (Table 1) summarizes the most important findings and provides information on the cohort of patients included in the study method of sample acquisition (stool versus biopsy) and methods used to classify their microbiomes. The exact characteristic profile of the ASD patient population cannot be determined, and there are some data showing contradicting results. This may explain the complexity and challenges that scientists face when pursuing ASD research. For example, this can possibly be attributed to a diverse patient population that varies in geographical location, diet, age, lifestyle, and antibiotic usage [54,55]. Additionally, the differences can be attributed to a small cohort size, sample acquisition method, sequencing technique, and software utilized [41,56]. Several studies point to Bacteroidetes and Firmicutes as phyla of importance, where their ratio is altered, with an increase in the former and decrease in the latter in the stool of the ASD population [41,44]. Of importance is the fact that the majority of the species in the Bacteroidetes phylum produce PPA [54,57]. Other studies showed that there was a significant increase in *Bacteroides*, *Desulfovibrio*, and *Clostridium* at the genus level in stool, all of which are PPA producers [41,42,44,58], and an elevated presence of *Bacteroides* is strongly corelated with an increase in PPA in patient stool samples [55]. The species most elevated in the *Bacteroides* genus include *B. uniformis*, *B. vulgatus*, and *P. distasonis* [41]. In addition to PPA, *Desulfovibrio* also produces Lipopolysaccharides (LPS) and hydrogen sulfide, which can have toxic effects [59]. Increases in both *Clostridia* and *Desulfovibrio* were found to be correlated with the use of antibiotics, and due to common comorbidities, ASD patients are prescribed antibiotics more often than the general population [60]. In the case of *Clostridia*, as it is a sporulating microbe, the population that was depleted after a course of antibiotics can be quickly replenished from spores [61]. Although *Desulfovibrio* does not produce spores, it is resistant to common antibiotics (such as cephalosporins) prescribed for ear infections that have a large prevalence in the ASD patient population [62]. Additionally, in a study where ASD patients were treated with antibiotics with high activity against Clostridium, their symptoms were significantly improved; however, the symptom improvement regressed after the conclusion of antibiotic treatment. It was hypothesized that the regression was caused by the reemergence of Clostridia from spores [63]. Additionally, an increase in PPA-producing *Faecalibacterium prausnitzii* was observed [41], alongside the *Sutterella*, *Lactobacillus*, *Roseburia*, *Enterobacter*, and *Akkermansia* genera [20,64]

The phyla Actinobacteria and Firmicutes were found to be significantly decreased in the stool of ASD groups based on several studies [41,44]; at the genus level, this includes *Actinomyces*, *Corynebacterium*, *Bifidobacterium*, *Ruminococcus*, *Streptococcus*, *Dialister*, *Fusobacterium Lachnospira*, and *Turicibacter* [20,44,65]. *Bifidobacterium* was consistently found by many studies to be at a lower abundance in ASD patients [20,44,65]. *Bifidobacterium* was found by several studies to have autoinflammatory effects, as well as the ability to regulate microbial composition [44,56,64,65,66,67,68]. 

In samples obtained from duodenal biopsies, *Burkholderia, Oscillospira*, *Actinomyces, Neisseria, Peptostreptococcus*, and *Ralstonia* are significantly elevated, while *Neisseria, Devosia, Prevotella, Bacteroides*, and *Streptococcus* are decreased in the ASD group vs. control [69]. Finally, in samples that originated from ileal and cecal biopsies, the order Clostridiale and families *Lachnospiraceae*, *Ruminococcaceae, Alcaligenaceae*, and *Methylobacteriaceae* are significantly elevated [70,71].

**Figure 1:**
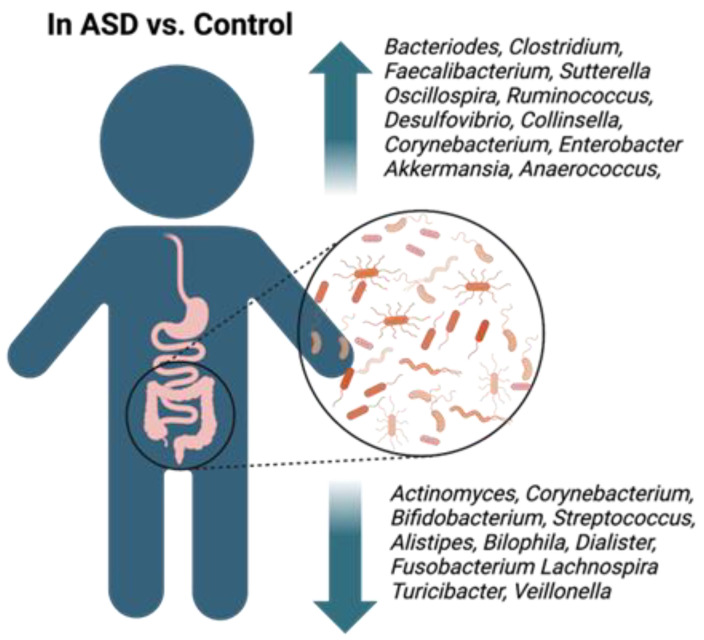
Summary of microbiota shift in ASD stating which bacterial genera had elevated or diminished levels (ASD versus age-matched controls) across the studies cited in Table 1. Created with Biorender.com.

A study by Li et al., where the microbiota of both children afflicted with ASD and their mothers were examined and compared to neurotypical controls, determined that Proteobacteria, Alphaproteobacteria, *Moraxellaceae*, and *Acinetobacter* were elevated in mothers with children afflicted with ASD. Also, it was determined that there was a clear correlation of bacteria present in mothers and their offspring, possibly due to vertical transfer. However, ASD children exhibited unique bacterial composition with an increase in *Alcaligenaceae*, *Enterobacteriaceae*, and *Clostridium* [72]. In a study by He et al. involving 40 ASD subjects with constipation (a common comorbidity in ASD), there was an increase in *Ruminococcaceae_UCG_002*, *Erysipelotrichaceae_UCG_003*, *Phascolarctobacterium*, *Megamonas*, *Ruminiclostridium_5*, *Parabacteroides*, *Prevotella_2*, *Fusobacterium*, and *Prevotella 9* and a decrease in *Anaerostipes*, *Lactobacillus*, *Ruminococcus_gnavus_group*, *Lachnosp raceae_NK4A136_group*, *Ralstonia*, *Eubacterium_eligens_ group*, and *Ruminococcus 1.* Figure 1 summarizes the recent findings, stating which bacteria were most commonly elevated or had diminished levels in the ASD group across studies.

**Table 1:** An overview of prominent studies aiming to elucidate the shift in gut microbiota in ASD patients. The author, year of publication, shift in specific microbiome (decrease or increase), and other prominent findings are included.

Author Year	Study Design				Change in ASD vs. Control		Other Findings
	Number of Participants	Age (Years)	Sample Source	Assessment Type	Increase	Decrease	
					P: Phylum O: Order F: Family G: Genus S: Species		
Coretti et al., 2018 [41]	ASD: 11 CON: 14	2–4	Stool	V3–V4 16S rRNA Illumina Miseq System	P: Bacteroidetes, Parabacteroidetes G: *Bacteroides*, *Faecalibacterium* *Oscillospira*, *Ruminococcus*	P: Actinobacteria G: *Actinomyces*, *Corynebacterium*, *Bifidobacterium*	Increased BA and PPA in ASD
Finegold et al., 2010 [44]	ASD: 33 CON: 15 (including 7 siblings of ASD and 8 nonrelated subjects)	2–14	Stool	bTEFAP FLX sequencer	P: Bacteroidetes, Proteobacteria G: *Desulfovibrio*, *Turicibacter* *Bacteroides Parabacteroides* S: *Desulfovibrio piger*, *Desulfovibrio Desulfovibrio intestinalis*, *Bacteroides vulgatus*	P: Firmicutes Actinobacteria G: *Weissella*, *Costridium*, *Actinomyces*, *Corynebacterium*, *Bifidobacterium*, *Ruminococcus Streptococcus*, *Dialister* S: *Dialister invisus*, *Bifidobacterium longum*, *Clostridium leptum*	Very high level of *Bacteroides* in severe cases of ASD
Parracho et al., 2005 [58]	ASD: 58 CON: 22 (12 siblings of ASD and 10 not related)	ASD: 3–16 CON: 2–13	Stool	FISH 16S rRNA oligonucleotide probes	S: *Clostridium histolyticum*		A high portion of the ASD group had GI issues
Strati et al., 2017 [20]	ASD: 40 CON: 40	4–17	Stool	V3–V5 16S rRNA. GS FLX + system	G: *Collinsella*, *Corynebacterium*, *Dorea*, *Lactobacillus*	G: *Alistipes*, *Bilophila*, *Dialister*, *Parabacteroides*, and *Veillonella*	ASD altered microbiota, constipation is an important factor
De Angelis et al., 2013 [42]	ASD: 10 CON: 10 siblings	4–10	Stool	bTEFAP 454 FLX Sequencer	P: Bacteroidetes, G: *Bacteroides Clostridium Roseburia Enterobacter Akkermansia*	P: Fusobacteria, Verrucomicrobia G: *Eubacterium*, *Fusobacterium*, *Lachnospira*, *Turicibacter*, *Bifidobacterium*	Increase in PPA and AA
Wang et al., 2020 [65]	ASD: 26 CON: 24	3–9	Stool	V1-V2 16S rRNA Illumina HiSeq sequencer	F: Rikenellaceae, G: *Ruminococcus*, *Victivallales Oscillospira*, *Odoribacter*, *Cetobacterium*,	P: Actinobacteria O: Bifidobacteriales, F: *Bifidobacteriaceae Veillonellaceae*, G: *Bifidobacterium*, S: *B. adolescentis*, *B. longum*	Decrease in PPA in ASD *Odoribacter*: common SCFA producer
Li et al., 2019 [55]	ASD: 59 children and their mothers CON: 30 children and their mothers	Children: 2–10 Mothers: 26–42	Stool	V1-V2 16S rRNA Illumina HiSeq sequencer	Children- G: *Enhydrobacter*, *Chryseobacterium*, *Streptococcus*, *Acinetobacter*, *Clostridium* S: *Acinetobacter rhizosphaerae*, *Acinetobacter johnsonii* Mothers-F: *Moraxellaceae Enterobacteriaceae* G: *Acinetobacter*	Children-S: *Prevotella* *melaninogenica* Mothers- G: *Faecalibacterium*	Assessment of mother–child gut microbiome profile. There is a clear correlation; however, a unique bacteria profile is still present in ASD children.
Kushak et al., 2017 [69]	ASD: 21 CON: 19 Both ASD and CON with GI symptoms	ASD: 14.43 ± 1.07 CON: 16.05 ± 1.25	Duodenum, endoscopic biopsy	16S rRNA 454 FLX Sequencer	G: *Burkholderia*, *Oscillospira*, *Actinomyces*, *Neisseria*, *Peptostreptococcus*, *Ralstonia*,	G: *Neisseria*, *Devosia*, *Prevotella*, *Bacteroides*, *Streptococcus*	Differences in bacteria associated with disaccharidase activity
Williams et al., 2011 [71]	ASD: 15 CON: 7 Both ASD and CON children had GI issues	3–6	Biopsy of ileal and cecal tissues	V2 16S rRNA 454 FLX Sequencer	O: Clostridiale F: *Lachnospiraceae*, *Ruminococcaceae*, *Alcaligenaceae*, *Methylobacteriaceae*	P: Bacteroidetes	Deficits in gene expression involved in carbohydrate digestion and transport
Williams et al., 2012 [70]	ASD: 15 CON: 7	3–5	Biopsy of ilium and cecum	V2 16S rRNA GS FLX sequencer	High level of species from *Sutterella* genus		*Sutterella* 16S rRNA in ASD group and absent in control
Adams et al., 2011 [66]	ASD: 58 CON: 39	ASD: 6.91 ± 3.4 CON: 7.7 ± 4.4	Stool	The Vitek^®^2 identification cards and Vitek 2 system	G: *Lactobacillus*, *Bacillus* spp.	G: *Bifidobacterium*, *Enterococcus* Species: *Enterobacter cloacae*	Decrease in SCFAs (lower SCFAs due to higher absortion/lower intake of fibers)
Tomova et al., 2015 [73]	ASD: 10 CON: 10 Siblings of ASD: 9	ASD: 2–9 CON:2–11 Sib.: 5–17	Stool	RT-PCR	*Clostridia* cluster l, *Desulfovibrio*	P: *Bacteroidetes*	Fecal TNFα increased in stool. Correlation between the amount of *Desulfovibrio* present and autism severity
Wang et al., [74]	ASD: 23 ASD siblings: 22 CON (unrelated): 9	ASD: 10.2 ± 0.75 CON: 9.5± 1.25 Sib.: 12 ± 1	Stool	RT-PCR	S: *Clostridium difficile*	S: *Akkermansia muciniphila*, *Bifidobacterium* spp.	Lower abundance of *Akkermansia muciniphila* is suggestive of changes in the mucosal barrier
David et al., 2021 [56]	ASD: 60 CON: 57 (siblings)	2–11	Stool	16S rRNA V4 Illumina MiSeq	G: *Bacteroides*, *Ruminococcus*, *Anaerococcus*	F: *Lachnospiraceae* G: *Desulfovibrio*, *Bifidobacterium*	Unique crowdsourcing recruitment of subjects.
Kang et al., 2013 [75]	ASD: 20 CON: 20	2–16	Stool	V2/V3 16S bTEFAP FLX Sequencer	G: *Akkermansia* present at very high level	P: Proteobacteria, Verrucomicrobi, G: *Veillonellaceae*, *Prevotella*, *Coprococcus*	Less diverse gut microbial composition in ASD
Finegold et al., 2017 [18]	ASD: 33 CON: 13	2–9	Stool	Anerobic bacteria culture. ABI 3130	Increase in *Clostridium*		Increase in *C. perfringens* beta2-toxin gene in ASD vs. control
Song et al., 2004 [76]	ASD: 15 CON: 8	Not specified	Stool	TaqMan RT-PCR 16S rRNA	Increases in Clostridium 46-fold: *C. bolteae* 9.0-fold: cluster I 3.5-fold: cluster XI		Study focused on *Clostridium*
Zhang et al., 2018 [77]	ASD: 35 CON: 6	ASD: 4.9 ± 1.5 CON: 4.6 ± 1.1	Stool	16S rRNA (V3–V4) Illumina HiSeq	P: Bacteroidetes G: *Sutterella*, *Odoribacter*, *Butyricimona*	P: Firmicutes Genus: *Veillonella*, *Streptococcus*	ASD group was characterized by increase in constipation
Son et al., 2015 [78]	ASD: 59 CON: 44 (siblings of ASD)	ASD:4–18 CON:7–14	Stool	V1V2 and V1V3 of 16S rRNA Illumina HiSeq	No difference found	No difference found	ASD group was characterized by increase in constipation
Wang et al., 2013 [79]	ASD: 23 CON: 31	Not specified	Stool	RT-PCR	G: *Sutterella* S: *Ruminococcus torques*		Focused on *Sutterella*
Jendraszak et al., 2021 [67]	ASD: 33 CON: 16 Allergies: 24	ASD: 4–6 CON: 3–9 ALG: 4–9	Stool	Microbial culture and RT-PCR		G: *Klebsiella*, *Bifidobacterium*	Probiotic use helps stabilize microbial composition
He et al., 2023 [40]	ASD: 40 CON: 40	ASD: 5.3 ± 1.34 CON: 5.83 ± 1.28	Stool	V3-V4 of the 16S rRNA Illumina HiSeq 2500	*Ruminococcaceae_UCG_002*, *Erysipelotrichaceae_UCG_003*, *Phascolarctobacterium*, *Megamonas*, *Ruminiclostridium_5*, *Parabacteroides*, *Prevotella_2*, *Fusobacterium*, *Prevotella 9*	*Anaerostipes*, *Lactobacillus*, *Ruminococcus_gnavus_group*, *Lachnospiraceae_NK4A136_group*, *Ralstonia*, *Eubacterium_eligens_ group*, *and Ruminococcus_1*	Children enrolled in this study suffered from constipation. Significant increase in SCFAs in the ASD group

### 3.3. Gut–Brain Axis

The gut–brain axis, often referred to as GBA, is a form of complex bidirectional communication between the central nervous system and the gastro-intestinal tract [65,80]. A focus on the gut microbiota and their metabolites in GBA has recently occurred. The modulation can be either direct (anatomical) via the vagus nerve (10th cranial nerve) and enteric nervous system or indirect with the involvement of metabolic, immune, and endocrine signaling pathways [35,81,82]. In past decades, research showed that the microbiota are among the key players that can influence virtually all aspects of the GBA [38,83]. One of the first sets of studies showing the involvement of microbiota in the GNS involved germ-free animals, in which their brains were altered in comparison to the non-germ-free control; additionally, other studies show that the administration of specific strains of microbes can alter animal behavior [83,84,85]. 

In the neurologic pathway, the vagus nerve and enteric nervous system can be directly affected by molecules produced by microbiota acting as neurotransmitters such as GABA, histamine, norepinephrine, acetylcholine, serotonin, dopamine, and melatonin [38,81,86,87,88]. On the other hand, the autonomic nervous system can modulate the activity of enteric neurons, smooth muscle cells, epithelial cells, and immune cells, which is responsible for the modulation of gut motility and permeability, mucus production, secretion of bile, intestinal osmolality, and fluid control [80,89].

In the metabolic pathway, the microbiome synthesizes many metabolites that can enter the systemic circulation, act on distant parts of the body, and modulate the behavior of many tissue and cell types including the brain [82]. For instance, SCFAs are utilized by colonic cells as an energy source, can be metabolized in the liver, or can cross the blood–brain barrier (BBB) and exert its effect on neurons and glial cells [35,41,90]. The exact pathways in which SCFAs exert their effects on the brain remain largely unknown. However, evidence shows that the direct effect of SCFAs on the brain is mainly exerted through two mechanisms: (1) through activation of GPR41 (free fatty acid receptor 3, which is expressed in the brain and BBB at high levels) and (2) through histone deacetylase (HDAC) inhibition in a dose-dependent manner [35,91,92,93,94]. The GPR41 receptor is activated by AA, BA, and PPA; however, the most potent activator of GPR41 is PPA [30,95]. The binding of SCFAs to GPR41 evokes a complex biological response [96]. GPR41 can be coupled to Gαi/o and evoke downstream effects including a decrease in cyclic adenosine monophosphate (cAMP), an increase in intracellular calcium concentration, and ERK1/2 activation [97]. In an in vitro experiment, it was also shown that differentiation of neural stem cells in media with a high concentration of PPA leads to overexpression of GPR41 in astrocytes, a decrease in PTEN, and an increase in Akt phosphorylation [3,98]. Histone modification is a form of epigenetic regulation that plays a large role in the nervous system’s development and homeostasis, and one of the most important modifications is acetylation. The acetylation of histones is a dynamic state regulated by two types of enzymes: acetyltransferases and HDACs [96]. PPA and BA are able to inhibit callas I and II and some of the III HDACs. 

**Figure 2:**
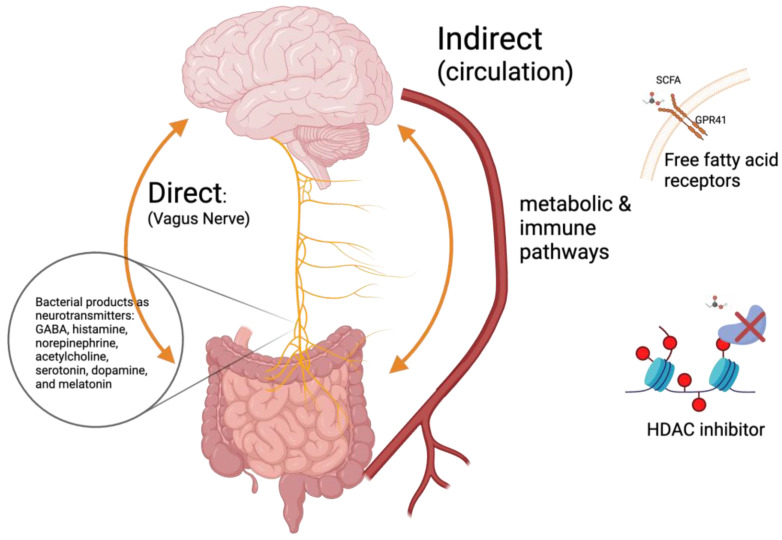
A summary of the involvement of SCFAs alongside other microbial metabolites in the GBA. Created with Biorender.com.

Finally, in the immune pathway, microbiota and their products modulate immune cells—either those residing in the vicinity of the GI tract or via systemic circulation of metabolites [83]. The effect of microbial metabolites, in particular of SCFAs, is exerted on both the adaptive and innate immune system, where they affect differentiation, migration, and the overall population of various cell types including T cells, macrophages, and innate lymphoid cells [99]. SCFAs downregulate the production of nuclear factor-κB (NF-κB) and tumor necrosis factor α (TNF-α) in lymphocytes and monocytes [100]. SCFAs also modulate the release of anti-inflammatory cytokines (mainly Il-10) [101]. SCFAs, via the modulation of GPR41, are important in the maturation and homeostasis of microglia. However, the anti-inflammatory effect of SCFAs is concentration- and tissue-specific [99]. Figure 2 summarizes the pathways in the GBA affected by microbial metabolites.

### 3.4. In Vivo Effect of SCFAs in Adult ASD-Animal Model

Several well-designed studies aimed to establish the effects of microbiota from ASD patients or microbial products directly (especially PPA and LPS) on rodents. Table 2 contains a summary of recent prominent studies. Sharon et al. used germ-free mice (GF) that were grafted with microbiota obtained from either ASD patients or typically developing (TD) control subjects [102]. In the group to which microbiota from ASD patients were grafted, there was a significant shift in behavior resembling that found in ASD, suggesting that the microbiota alone are sufficient to produce ASD-like symptoms in a rat model. Those hallmark behaviors included a significant decrease in locomotion (evidenced by an open field test) and communication (evidenced by a vocalization test), alongside an increase in repetitive behaviors (evidenced by a marble burring test) [102,103]. Interestingly, the symptoms were more prominent in the male rats than the female rats used in the study, which resembles the gender disparity seen in the ASD population, with a significantly higher ratio of males being affected [104]. The grafted microbiome in the ASD group had an increased population of bacteria in the following taxa: Bacteriodetes, b-Proteobacteria, Lactobacillales, Clostridiaceae, and Enterobacteriaceae—which matches other published findings [20,41,44,64]. It was also found that the metabolic profile was altered between both groups, and genes relevant to brain function were alternatively spliced. 

In two studies conducted by MacFabe et al., adult mice were injected with the microbial metabolite PPA [105,106]. In the treated rodents, there was an alteration in behavior characterized by lesser sociability, increased focus on particular objects, and an increase in respective behavior. At the molecular level, PPA-treated mice were characterized by an increase in oxidative stress, microglia, and astrocyte activation (an increase in GFAP). 

In two additional studies, high doses of PPA were delivered orally (in one of the studies, this was in conjunction with ampicillin treatment and, in another, with clindamycin). An increase in catalase and lipid peroxidation was observed in the first study, while potassium and glutathione levels were decreased in the brain, suggesting that oral ammonization of a high dosage of PPA has a neurotic effect [107]. In the second study, there was a shift in the microbiota, an increase in the Clostridium species, and an increase in Na+/Mg^2^+ and glutamate/GABA ratios (in the brain) in PPA- and clindamycin-treated golden Syrian hamsters vs. the control [108].

In a study published by Lobzhanidze et al., a single and relatively low dose of buffered PPA (175 mg/kg) delivered via intraperitoneal injection significantly altered the behavior of young Wistar rats versus the vehicle-treated control. Treated animals spent significantly less time with unfamiliar rats, signifying decreased interest in social stimuli. Histological evaluation of the amygdala neurons and glia cells show a significant increase in glia cells in the PPA-treated rats with evidence of swollen or proliferating astrocytes and activated microglia. Additionally, a slight decrease in neurons in the amygdala was detected [109]. 

Finally, prenatal and postnatal injection of PPA and LPS into Long–Evans rats altered their behavior and induced delays in eye opening (a physical milestone in mice). In the open field test, mice exposed to PPA prenatally exhibited more anxiety, signified by a decreased amount of time spent in the center of the open field. PPA delivered pre- and postnatally (in the same animals) increased repetitive behavior, suggesting that PPA exposure prenatally (in utero) and postnatally can evoke ASD-like behaviors in a rodent model [110]. 

This compelling body of evidence indeed shows that SCFAs and microbiota from ASD patients can evoke ASD-like symptoms. Several clinical trials involving fecal transfer showed improvements in ASD symptoms [111].

**Table 2:** An overview of prominent studies aiming to elucidate the effect of PPA in adult ASD-animal models. The study design (animal type, sample size, and type of treatment) is described, alongside the most important outcomes.

Author Year	Study Design			Outcomes
	Animal	Sample Size	Treatment	
Sharon et al., 2019 [102]	Mice: Germ-free C57BL/6J weanlings (3–4 weeks of age)	16 donor fecal samples 9 animals colonized by bacteria from each donor sample	GF mice grafted with gut microbiota from ASD and TD control subjects	Microbiota from ASD altered the behavior of mice: increased repetitive behavior, decreased locomotion, and decreased communication. It also induced alternative splicing of genes in the mice brain in ASD vs. TD control. Differences in the metabolome profile.
MacFabe et al., 2007 [105]	Adult male Long–Evans rats (~75 days old)	Total of 74 rats across groups Group sizes 6–9 animals	Infusion with PPA. Low: 4.0 μL of a 0.052 M solution; high: PPA (4.0 μL of a 0.26 M solution. Controls: PBS or propanol	PPA treatment: increase in oxidative stress markers. Altered behavior (repetitive dystonic behaviors, hyperactivity, and turning behavior). Increased reactive astrogliosis (GFAP immunoreactivity) and activated microglia (CD68 immunoreactivity).
Meeking et al., 2020 [112]	Adult male Long–Evans	Total of 35 rats across groups	7 days, twice a day, 4 h apart, infusion of buffered PPA (low dose 0.052 M or high dose 0.26 M, pH 7.5, 4 μL/infusion) control: phosphate buffered saline (PBS, 0.1 M)	PPA-treated rats exhibited more locomotive activity, stereotypic behavior, and nose pokes versus control, which are associated with a rat model of ASD. The symptoms were dose-dependent and increased with consecutive treatments.
De Theije et al., 2014 [113]	BALB/C mice from Charles River laboratories	8 pups in treatment group and 11 in control	Dams treated at gestational day 11 with 600 mg/kg of valproic acid (VPA). Pups weaned at P21. Behavioral experiments performed at P28, after which they were sacrificed. VPA treatment during gestation is well established in the animal model of ASD.	An increase in cecal levels of BA in in utero VPA-treated pups vs. control. A decrease in *Bacteroidales* (order) and increase in *Clostridiales* (order) in VPA vs. control. Increased neutrophil infiltration in the intestine.
MacFabe et al., 2011 [106]	Adolescent (41 ± 4 days) Long–Evans male	20 and 17 animals in PPA and control groups, respectively	Intracerebroventricular injection of 4 μl of 0.26 M buffered PPA prior to each test session	PPA vs. control group characterized by activation of microglia and astrocytes, lesser sociability, and a focus on particular objects in a group of objects.
El-Ansary et al., 2015 [107]	Male Western albino rats	6 animals in each group	PPA: 250 mg/kg body weight/day (orally) Ampicillin: 50 mg/kg for three weeks	Treatment with PPA and ampicillin led to an increase in catalase activity and lipid peroxidation, while glutathione and potassium levels were decreased in comparison to the control group.
El-Ansary et al., 2018 [108]	Young male golden Syrian hamsters	10 animals in each group	PPA: 250 mg per kg of body weight (BW) (oroigastric) Clindamycin: 30 mg single dose	An increase in *Candida albicans* and *Clostridia* in PPA and clindamycin groups. An increase in Na+/Mg2+ and glutamate/GABA ratios.
Lobzhanidze et al., 2019 [109]	Adolescent male Wistar rats (P30–35)	15 animals in each group	Single injection of buffered PPA with a dose of 175 mg/kg	In the PPA vs. control groups, the number of neurons was decreased, while the number of glial cells was increased in the amygdala. Also, both microglia and astrocytes were activated, and neurons exhibited signs of apoptosis. The behavioral changes include decreased sociability (a decrease in the amount of time and number of encounters with unfamiliar rats).
Foley et al., 2014 [110]	Long–Evans rats, offspring treated in utero and postnatal	8 to 11 animals in each group	Prenatal administration of PPA (500 mg/kg,) and LPS (50 μg/kg). Postnatal PPA administered at PPA (500 mg/kg)	Treatments (both prenatal and postnatal) altered the behavior of rodents to autism-like behavior. PPA-treated rats spend less time in the center of the open field and exhibited increased anxiety. Treatment induced delays in eye opening.

## 4. Conclusions

In recent years, there has been staggered growth in the appreciation of the role of microbiota in many neurological conditions [114,115], and in some cases, the administration of specific microbiota may have therapeutic effects [116]. In the case of ASD, it has been repeatedly shown that a shift in the microbiota is present versus the ND control, with an evident increase in bacteria that produce PPA, such as *Bacteroides*, *Desulfovibrio*, and *Clostridium.* Rodent models of ASD showed that the grafting of the microbiome from ASD patients to GF mice can alter their behavior. Furthermore, the models showed that direct administration of PPA can evoke ASD-like symptoms in rodents, as well as inflict molecular changes in the brain that are associated with ASD. Those findings contribute strong evidence that microbiota shift and the resulting changes in SCFAs, particularly PPA, may contribute to the development of ASD, while the avoidance of PPA and regulation of the microbiome shift may contribute to lowering the risk of ASD development or to the improvement of ASD symptoms. 

Some of the limitations of this review stem from the heterogenicity in both ASD and the sources from which the samples were obtained. ASD has a very wide range of symptoms, comorbidities, and factors contributing to its development (both genetic and environmental); thus, it is often challenging to isolate a uniform set of microbes contributing to ASD development. Additionally, the sample sizes tend to be small, and many confounding factors such as diet or lifestyle additionally contribute to heterogenicity. 

Testing of the gut microbiota for signs of dysbiosis in patients diagnosed with ASD, prescribing probiotics, or fecal transplants may potentially lead to the improvement of ASD symptoms. 

Overall, as we have shown in this comprehensive literature review, there is a large body of data that clearly associates SCFAs, especially PPA, with alterations in neurodevelopment and social behavior in animals consistent with those seen in humans with ASD. Our research team is pursuing a unique study that is focused on investigating the effect of PPA on pregnant mice and their offspring. This ongoing study should provide critical data toward understanding possible alterations in fetal neurodevelopment during pregnancy and the development of ASD in newborns.
